# Upregulation in Inflammation and Collagen Expression in Perirenal but Not in Mesenteric Adipose Tissue from Diabetic Munich Wistar Frömter Rats

**DOI:** 10.3390/ijms242317008

**Published:** 2023-11-30

**Authors:** Elena Vega-Martín, Daniel González-Moreno, Marta Sanz-Gómez, Ana Karen Guzmán-Aguayo, Francisco Javier Manzano-Lista, Angela Schulz, Isabel Aránguez, Reinhold Kreutz, María S. Fernández-Alfonso

**Affiliations:** 1 Instituto Pluridisciplinar and Facultad de Farmacia, Universidad Complutense de Madrid, 28040 Madrid, Spainanakguzm@ucm.es (A.K.G.-A.);; 2 Department of Clinical Pharmacology and Toxicology, Charité-Universitätsmedizin Berlin, 10117 Berlin, Germany; 3 Instituto de Investigación Sanitaria del Hospital Clínico San Carlos (IdISSC), 28040 Madrid, Spain

**Keywords:** chronic kidney disease, diabetes, perirenal adipose tissue, mesenteric adipose tissue, experimental model, Munich Wistar Frömter rat

## Abstract

Perirenal adipose tissue (PRAT) surrounding the kidney is emerging as a player and novel independent risk factor in diabetic kidney disease (DKD); DKD is a complication of diabetes and is a major cause of increased cardiovascular (CV) risk and CV mortality in affected patients. We determined the effect of diabetes induction on (i) kidney and CV damage and (ii) on the expression of proinflammatory and profibrotic factors in both the PRAT and the mesenteric adipose tissue (MAT) of Munich Wistar Frömter (MWF) rats. The 16-week-old male MWF rats (n = 10 rats/group) were fed standard chow (MWF-C) or a high-fat/high-sucrose diet for 6 weeks together with low-dose streptozotocin (15 mg/kg i.p.) at the start of dietary exposure (MWF-D). Phenotyping was performed at the end of treatment through determining water intake, urine excretion, and oral glucose tolerance; use of the homeostatic model assessment–insulin resistance index (HOMA-IR) evidenced the development of overt diabetes manifestation in MWF-D rats. The kidney damage markers *Kim-1* and *Ngal* were significantly higher in MWF-D rats, as were the amounts of PRAT and MAT. A diabetes-induced upregulation in *IL-1*, *IL-6*, *Tnf-α*, and *Tgf-β* was observed in both the PRAT and the MAT. *Col1A1* was increased in the PRAT but not in the MAT of MWF-D, whereas *IL-10* was lower and higher in the PRAT and the MAT, respectively. Urinary albumin excretion and blood pressure were not further increased by diabetes induction, while heart weight was higher in the MWF-D. In conclusion, our results show a proinflammatory and profibrotic in vivo environment in PRAT induced by diabetes which might be associated with kidney damage progression in the MWF strain.

## 1. Introduction

Chronic kidney disease (CKD) is a common progressive, incurable condition which will soon become the fifth leading cause of death worldwide [1]. The rising burden of CKD (9.1% to 13.4% of the global population [2]) is demonstrated by its growing impact on total mortality, as well as on the associated costs, with CKD-related care being the foremost driver of healthcare costs for most European countries [3]. This surge is due to the global rise in some key risk factors, such as diabetes mellitus (DM, 537 million adults), obesity, and metabolic syndrome [4].

Major long-term macro- and microvascular complications of DM and CKD include endothelial dysfunction, blood pressure increase, and the development of arterial stiffness, leading to reduced coronary artery perfusion, increased cardiac workload, and chronic cardiac dysfunction [5,6]. Diabetic kidney disease (DKD) is a complication of diabetes and is a major cause of increased cardiovascular (CV) risk and CV mortality in these patients due to the fatal complications in diabetic cardiomyopathy or stroke [7].

DKD is a heterogeneous disease regarding its pathophysiology, histopathology, clinical manifestations, and rate of progression. The etiology of DKD is complex and multifactorial, involving both metabolic and hemodynamic factors; comorbidities, such as hypertension or obesity, affect its clinical course. In many cases, kidney dysfunction may not be primarily caused by DM, but it can develop or be promoted in the context of preexisting kidney damage [8].

Perirenal adipose tissue (PRAT), a visceral fat depot surrounding the kidney [9], is emerging as a player and novel independent risk factor in CKD patients [10,11,12,13,14,15,16]. Elevated PRAT thickness (measured by ultrasound) is associated with a decrease in the estimated glomerular filtration rate (eGFR) in hypertensive10, obese11, and diabetic patients [12,13,14,15,16], as well as those with CKD and CV disease progression [17,18]. The anatomical position of PRAT adjacent to the kidney and its different developmental program compared to other fat depots [19] makes the PRAT a possible bystander in CKD. Therefore, a possible paracrine deleterious influence of PRAT on the kidney should not be excluded. Although potential mechanisms have been suggested [19], there is little evidence for possible PRAT dysfunction in CKD and its deleterious paracrine effect.

The Munich Wistar Frömter (MWF) rat is a genetic model of spontaneous and progressive non-diabetic albuminuria development [20] that mirrors several features that have been observed in patients with CKD [21,22]. In response to an inherited nephron deficit of 30–50%, this model shows glomerular hyperfiltration and develops progressive albuminuria, kidney injury (glomerulosclerosis and interstitial fibrosis), mild hypertension, and vascular disease with age: albuminuria (8-week-old rats), endothelial dysfunction (12-week-old rats), hypertension (14-week-old rats), arterial stiffness (16-week-old rats), and increased pulse wave velocity (22-week-old rats) [21,22,23,24,25,26,27,28].

In this context, we aim to determine the effect of diabetes induction on the expression of proinflammatory and profibrotic factors in PRAT in comparison with another visceral adipose tissue depot—the mesenteric adipose tissue (MAT). Moreover, we aim to analyze the impact of diabetes on the preexisting kidney and cardiovascular damage in the MWF strain. For this purpose, we have treated 16-week-old MWF rats, which already exhibited CKD and its CV complications, with low-dose streptozotocin (STZ) together with short-term exposure to a high-fat high-sucrose (HF/HS) diet; we analyzed the metabolic, kidney, and CV damage.

## 2. Results

### 2.1. MWF-D Showed an Increase in Body Weight and Adiposity after Treatment

**Figure 1:**
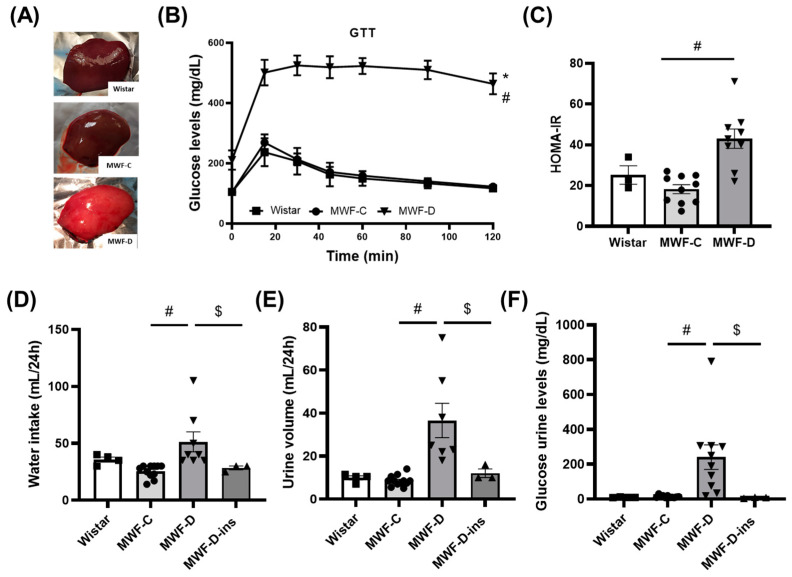
Glycemic parameters. (**A**) Gross anatomy of control rat liver (Wistar and MWF-C) and liver with steatosis (MWF-D). The fatty liver has a yellowish appearance. (**B**) Plasma glucose levels during glucose tolerance test (GTT), (**C**) HOMA-IR, (**D**) water intake, (**E**) 24 h urine volume, and (**F**) glycosuria in Wistar (■), MWF-C (⬤), MWF-D (▲), and MWF-D-ins (▼) rats. Data are expressed as the mean ± SEM of *n* = 3–10 animals per group. * *p* < 0.05 compared to Wistar; # *p* < 0.05 compared to MWF-C; $ *p* < 0.05 compared to MWF-D.

As shown in Table 1, MWF-C showed lower body weights compared to Wistar probably due to strain differences, as previously described [22,23]. The daily caloric intake was substantially higher in MWF-D fed the high-energy diet than in MWF-C. This was reflected in a significant body weight increase in the MWF-D group at the end of the 6-week treatment compared to MWF-C, associated with a significant increase in the amounts of PRAT and MAT (Table 1), as well as the development of fatty liver (Figure 1A).

### 2.2. MWF-D Developed Hyperglycemia, Glucose Intolerance, Insulin Resistance, Polydipsia, and Polyuria

MWF-D rats showed a significant increase in both fasting and postprandial blood glucose as compared to the MWF-C group. Fasting insulin levels were similar to the control strain, whereas postprandial insulin was significantly lower in MWF-D (Table 1).

To determine whether MWF-D had a worse glycemic control, a GTT was performed. As shown in Figure 1B, glucose administration induced a substantially greater increase in blood glucose in MWF-D than in the MWF-C, with peak blood glucose levels reaching a maximum at 15 min. Also, while MWF-C rats reached basal levels 2 h after glucose overload, MWF-D rats still had significantly higher blood glucose levels (>400 mg/dL) than baseline, showing a significant reduction in glucose tolerance in the MWF-D group. Moreover, MWF-D rats exhibited a significant increase in HOMA-IR (Figure 1C) compared to MWF-C rats, showing that this group also developed insulin resistance. 

The MWF-D group exhibited the classical features of diabetes, i.e., higher water intake (Figure 1D), higher urine volume (Figure 1E), and increased glycosuria (Figure 1F), whose increase was prevented by insulin treatment in the MWF-D-ins group (Figure 1D–F).

### 2.3. MWF-D Exhibited a Worsening in Kidney Damage

**Figure 2:**
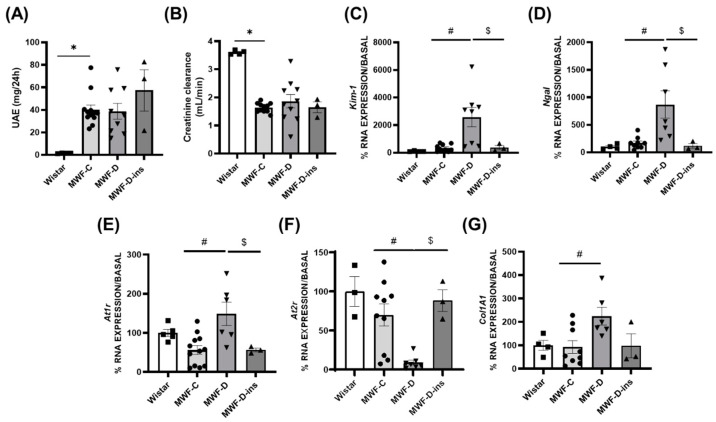
Kidney parameters in Wistar (■), MWF-C (⬤), MWF-D (▲), and MWF-D-ins (▼) rats. (**A**) Urinary albumin excretion (UAE); (**B**) creatinine clearance and kidney mRNA expression of (**C**) *Kim-1*, (**D**) *Ngal*, (**E**) *At1r*, (**F**) *At2r*, and (**G**) *Col1A1*. Data are expressed as the mean ± SEM of *n* = 3–10 animals per group. * *p* < 0.05 compared to Wistar; # *p* < 0.05 compared to MWF-C; $ *p* < 0.05 compared to MWF-D.

The relative left kidney weight was significantly increased in the MWF-D group compared to the MWF-C animals (MWF-C = 29.41 ± 1 mg/mm vs. MWF-D = 34.14 ± 1 mg/mm, *p* < 0.05; normalized by tibia length). No differences were observed between MWF-C and Wistar (35.33 ± 4.4 mg/mm). UAE (Figure 2A) was higher in MWF-C and MWF-D compared to Wistar rats, with no impact of diabetes or insulin treatment observed. Creatinine clearance (Figure 2B) was significantly decreased in MWF-C compared with Wistar (1.639 ± 0.05 vs. 3.60 ± 0.04 mL/min), with no changes in diabetes or insulin treatment.

Kidney expression of tubular damage markers *Kim-1* (Figure 2C) and *Ngal* (Figure 2D) was significantly higher in MWF-D compared to MWF-C. Moreover, expression of fibrosis relevant bystanders, such as the angiotensin AT_1_ receptor (*At1r*, Figure 2E) and collagen 1A1 (*Col1A1*, Figure 2G) were significantly higher in MWF-D compared to MWF-C, whereas expression of the protective angiotensin AT_2_ receptor (*At2r*, Figure 2F) was significantly reduced in the diabetic group.

Insulin treatment prevented the upregulation in tubular damage markers *Kim-1* and *Ngal* thus confirming that STZ does not exert tubular damage [29] at the doses used in this study and that the observed effects might be attributed to hyperglycemia.

### 2.4. Expression of Inflammatory and Profibrotic Factors in PRAT and MAT

**Figure 3:**
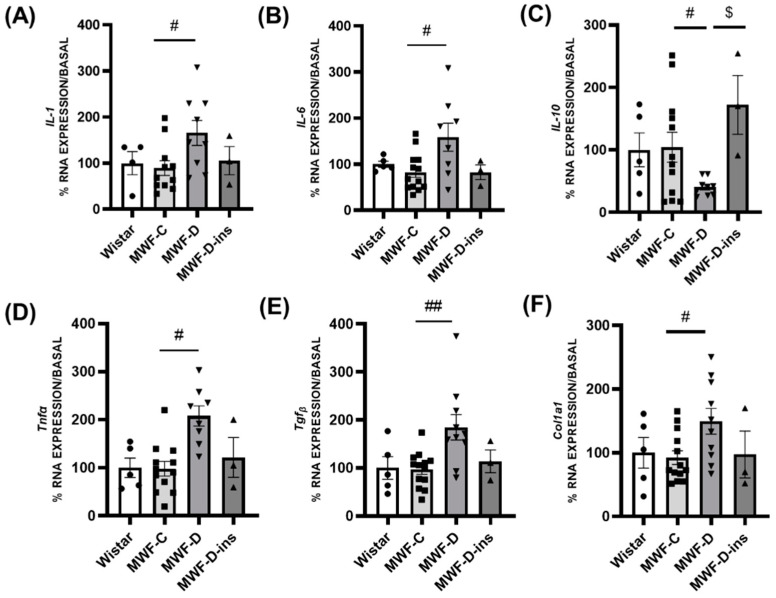
Perirenal adipose tissue mRNA expression of inflammatory and fibrotic markers in Wistar (■), MWF-C (⬤), MWF-D (▲), and MWF-D-ins (▼) rats. (**A**) *IL-1*, (**B**) *IL-6*, (**C**) *IL-10*, (**D**) *Tnf-α*, (**E**) *Tgf-β*, and (**F**) *Col1A1*. Data are expressed as the mean ± SEM of *n* = 5–12 animals per group. # *p* < 0.05 and ## *p* < 0.01 compared to MWF-C; $ *p* < 0.05 compared to MWF-D.

In the PRAT, the expression of proinflammatory *IL-1* (Figure 3A), *IL-6* (Figure 3B), and *Tnf-α* (Figure 3D), as well as profibrotic *Tgf-β* (Figure 3E) and *Col1A1* (Figure 3F) were significantly upregulated in the MWF-D group compared to the MWF-C group. The anti-inflammatory *IL-10* (Figure 3C) was downregulated by diabetes compared to MWF-C. No differences were observed between the MWF-C and MWF-D-ins groups, showing that diabetes-induced changes in expression were prevented through insulin treatment.

**Figure 4:**
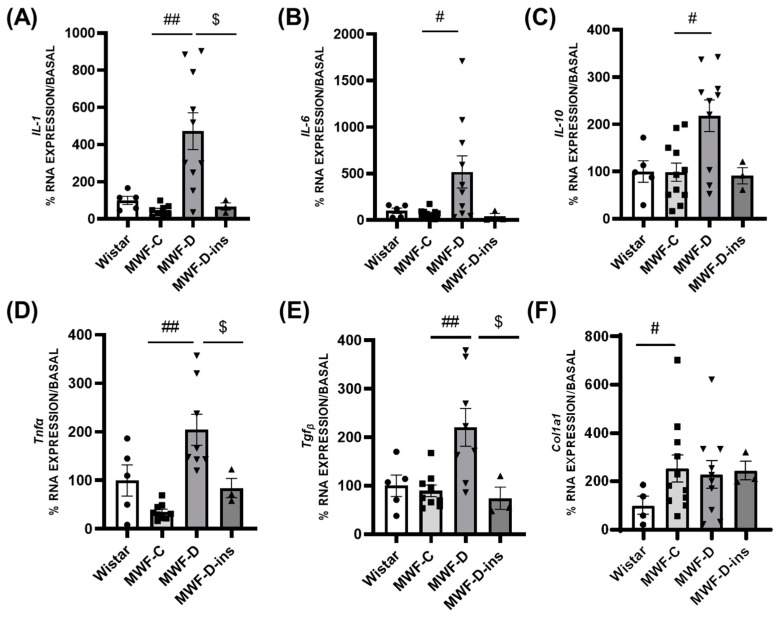
Mesenteric adipose tissue mRNA expression of inflammatory and fibrotic markers in Wistar (■), MWF-C (⬤), MWF-D (▲), and MWF-D-ins (▼) rats. (**A**) *IL-1*, (**B**) *IL-6*, (**C**) *IL-10*, (**D**) *Tnf-α*, (**E**) *Tgf-β*, and (**F**) *Col1A1*. Data are expressed as the mean ± SEM of *n* = 5–10 animals per group. # *p* < 0.05 and ## *p* < 0.01 compared to MWF-C; $ *p* < 0.05 compared to MWF-D.

In the MAT, the expression of *IL-1* (Figure 4A), *IL-6* (Figure 4B), *IL-10* (Figure 4C), *Tnf-α* (Figure 4D), and *Tgf-β* (Figure 4E) were significantly higher in the MWF-D group compared to the MWF-C group. This upregulation was prevented by insulin treatment. No differences were observed between the MWF-C and MWF-D in *Col1A1* (Figure 4F) expression.

### 2.5. Hemodynamic and Cardiac Parameters in MWF-D

Since DKD is associated with an increased CV risk, we ought to determine the effect of diabetes on hemodynamic and cardiac parameters in MWF-D. The SBP, DBP, and PWV values were similar between MWF-C and MWF-D, with no further effect of diabetes; however, these were significantly higher compared to those of Wistar rats (Table 2).

**Figure 5:**
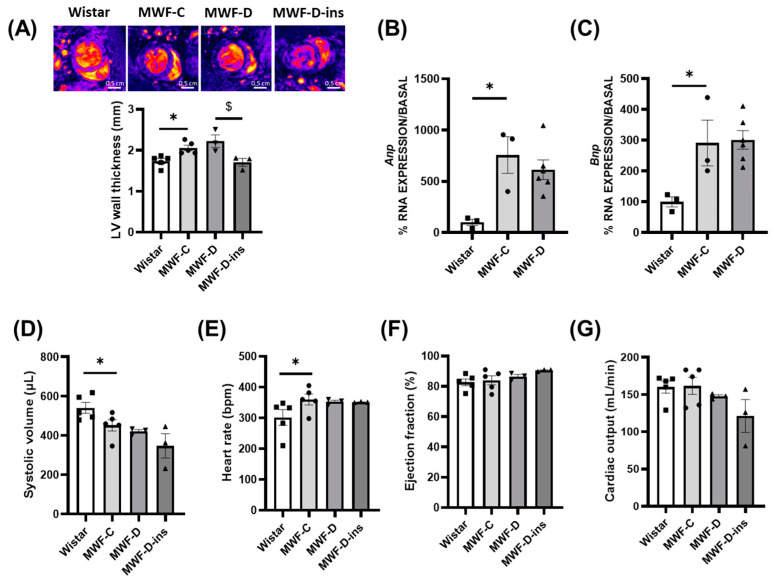
Cardiac function and structure in Wistar (■), MWF-C (⬤), MWF-D (▲), and MWF-D-ins (▼) rats. (**A**) Representative cross-section of the heart obtained by MRI during end diastole (upper panel) and quantitative analysis of LV free wall thickness (lower panel); (**B**) cardiac mRNA expression of *Anp*; (**C**) cardiac mRNA expression of *Bnp*; (**D**) systolic volume; (**E**) heart rate; (**F**) ejection fraction; (**G**) cardiac output. Data are expressed as the mean ± SEM of *n* = 3–5 animals per group. * *p* < 0.05 compared to Wistar; $ *p* < 0.05 compared to MWF-D.

Relative heart weight was significantly higher in MWF-D (MWF-C = 23.8 ± 1 mg/mm vs. MWF-D = 28.0 ± 1 mg/mm, *p* < 0.05; expressed by tibia length) and significantly lower in the MWF-D-ins group (24 ± 0.2 mg/mm; *p* < 0.05). In contrast, no effect of diabetes was observed in either LV wall thickness determined using MRI (Figure 5A) or cardiac expression of *Anp* (Figure 5B) and *Bnp* (Figure 5C).

The cardiac function, systolic volume (Figure 5D), and heart rate (Figure 5E) parameters were similar in both MWF-C and MWF-D. No differences between the groups were observed in the ejection fraction (Figure 5F) or cardiac output (Figure 5G).

## 3. Discussion

Here, we show for the first time that diabetes induces a net proinflammatory and profibrotic environment in PRAT from MWF-D rats. These rats developed glucose intolerance and insulin resistance, leading to a worsening of kidney damage that was independent of hypertension or albuminuria. 

MWF-D rats developed glucose intolerance, as well as an increase in the HOMA-IR index indicative of insulin resistance, together with polyuria, glycosuria, and polydipsia; these are characteristic symptoms of diabetes. There are no references in the literature with doses as low as 15 mg/kg i.p. in Wistar or Sprague Dawley rats. This is probably because of the increased sensitivity of the MWF strain to STZ, as described in Section 4. However, the observed metabolic features are consistent with those reported in other studies, both in rats treated with a STZ dose of 15 mg/kg (i.v.) [30,31,32], 25 mg/kg [33,34,35,36], 30 mg/kg (i.p.) [37,38,39,40,41,42,43], or two doses of 20 mg/kg [29], as well as in SD rats fed a high-fat, high-sucrose diet (34% sucrose, 42% calories from fat) [37]. In our opinion, this lower dose is advantageous because, in addition to the desired induction of diabetes, it has a low potential to cause undesirable direct dose-dependent toxicity, for example in the kidney. Accordingly, the level of hyperglycemia and the diabetic environment plays a more important role and strengthens the validity of our model.

The high energy/STZ rat is a widely used experimental model of diabetes which combines an initial administration of the β-cell toxin STZ with a high-fat and in some cases high-sugar diet for several weeks [37,44] (for review [29]). It is generally used as a type 2 (T2DM) diabetes model [29]; however, it is still a matter of debate whether it is a T1DM model or not [29,44,45,46]. In fact, depending on the STZ dose and the number of administrations, as well as the type of high energy diet used, it might be also considered a T1DM model [35,45,46]. The order of pathological events, with the STZ-induced β-cell failure occurring first, followed by the body weight increase in the subsequent weeks, better mimics a precedent T1DM coexisting with obesity; this is because obesity is more often seen in patients after their T1DM diagnosis. In contrast, T2DM often develops in patients with obesity after obesity maintenance [29]. In addition, although the MWF-D group did not lose weight (a typical feature of T1DM), it showed only a moderate increase in body weight, i.e., less than a 10% increase. This was probably compensated for by the short-term high-energy diet. MWF-D rats also show postprandial hypoinsulinemia and not the typical compensatory hyperinsulinemia to preserve normoglycemia observed in T2DM [29]. With these criteria, we believe that our model exhibits a significant T1DM component. In any case, it needs to be stressed that a central driver of kidney damage in diabetes is hyperglycemia, irrespective of whether it is T1DM or T2DM [8].

Adipose tissue expansion is tightly associated with adipose tissue dysfunction and ectopic fat accumulation in non-adipose tissues, such as the liver [47], as we observe in MWF-D probable due to de novo lipogenesis associated with the hyperglycemic milieu [48]. Although fatty liver is often considered a feature of T2DM, a recent meta-analysis shows that its prevalence in patients with T1DM is considerable [49]. Therefore, the development of a fatty liver in MWF-D should not be a reason to consider the model as T2DM and not as T1DM, as discussed above.

IL-10 plays a critical role in suppressing the inflammatory response by downregulating proinflammatory cytokines, such as IL-1, IL-6, and TNF-α [50]. The herein-reported imbalance between these cytokines in PRAT of MWF-D rats thus suggests a net inflammatory milieu in response to diabetes. Moreover, the upregulation in *Tgf-β* and *Col1A1* expression also indicates a profibrotic environment in PRAT, since TGF-β constitutes the main bystander in fibrotic processes stimulating collagen deposition [51]. In this context, a possible paracrine deleterious influence of PRAT on the CKD progression, characterized by fibrosis of renal parenchyma, aberrant deposition of collagen, and infiltration of inflammatory cells [52], should not be excluded.

The anatomical position of the PRAT, adjacent to the kidney, and its different developmental program compared other fat depots makes PRAT a possible bystander in CKD. In fact, recent studies have highlighted the key role of PRAT thickness as a novel independent risk factor for CKD in patients. Elevated PRAT thickness (measured by ultrasound) is associated with a decrease in the estimated glomerular filtration rate (eGFR) in patients with obesity [11] and diabetes [12,13,14,15,16], as well as those with CKD and CV disease progression [17,18]. The increased amount of PRAT in the MWF-D group was not found to be associated with a further increase in UAE or creatinine clearance compared to the MWF-C group; however, an upregulation in tubular injury markers *Ngal* and *Kim-1* was observed. Moreover, angiotensin II regulates TGF-β expression and collagen synthesis through the angiotensin receptor AT_1_R [53], balanced by AT_2_R which inhibits TGF-β and collagen deposition [54]; therefore, the upregulation in the *At1r* in the kidney, the upregulation in collagen expression, and the downregulation in the *At2r* indicate profibrotic changes that will further increase kidney fibrosis in this model. Interestingly, the PRAT has been also associated with an activation of the renin–angiotensin–aldosterone system [10]. To note, the abovementioned changes in kidney damage are not related to a possible STZ toxicity. Tubular toxicity has been reported at doses of 200 mg/kg or at repeated doses of 5 × 50 mg/kg [55]; however, a possible tubular damage at the dose of 15 mg/kg was excluded in the insulin-treated group. Therefore, we might conclude that the changes observed in kidney are specifically attributable to hyperglycemia. Moreover, we are describing early mechanisms of kidney damage progression due to short-term exposure to diabetes; these are not yet reflected in a further alteration of UAE or creatinine clearance.

In MAT, however, the upregulation in inflammatory cytokines might be compensated by the significant increase in *IL-10.* Moreover, *Col1A1* expression is not enhanced in MAT despite the increase in *Tgf-β*, suggesting that there is not an overt fibrotic response in this adipose depot yet. The differential expression of inflammatory and profibrotic factors between PRAT and MAT highlights the phenotypic differences between two adjacent adipose tissue depots, as previously reported for other adipose pads [56]. Note that our model reflects early mechanisms of adipose tissue dysfunction initiation due to a short-term exposure to diabetes. In this scenario, the lower inflammatory and profibrotic factor expression in MAT might be a compensatory mechanism of the dysfunction observed in PRAT and explain the absence of further hemodynamic alterations at this time. Nonetheless, this does not exclude the fact that inflammation in diabetic visceral adipose tissue is innocuous or inconsequential at later time points.

As described before, the MWF rat model develops progressive kidney injury, mild hypertension, and vascular disease with age, starting with early progressive albuminuria development at 8-weeks of age, followed by endothelial dysfunction, hypertension, arterial stiffness, and increased pulse wave velocity [21,22,23,24,25,26,27,28]. In this study, the high-energy/STZ model was developed at 16 weeks of age, at which kidney and cardiovascular damage is already established before diabetes induction. However, we did not observe a further increase in blood pressure values induced by diabetes in comparison with the already hypertensive MWF-C rats or a further increase in PWV at 22 weeks of age at the end point. The heart weight reveals (despite similar *Anp* and *Bnp* expression levels) a blood-pressure-independent increase in weight in response to diabetes, since it is prevented by insulin treatment. This observation supports the idea that our model reflects the early mechanisms of cardiac damage initiation due to short-term exposure to diabetes that is independent of further hemodynamic alterations. This is noteworthy and is consistent with the reduced systolic volume and increased heart rate, maintaining cardiac output. The ejection fraction is still preserved in our model at the time window of short-term diabetes exposure. Since heart enlargement is related to heart failure, with a preserved ejection fraction (HFpEF)—an entity that is becoming increasingly important in the cardiology field—we believe that future studies analyzing the MWF-D rat at an older age and under longer dietary treatments might shed light on the pathophysiological mechanisms of cardiorenal dysfunction. Interestingly, cardiorenal dysfunction has recently also been associated with alterations in the PRAT [17,18].

There are several limitations in the present study. First, this is an association study that does not demonstrate causality. It describes how diabetes was observed to induce a proinflammatory and profibrotic environment in the PRAT derived from MWF-D rats, and that this was seen to be associated with worsening in the preexisting kidney damage among MWF-C rats. However, the questions of whether this PRAT dysfunction caused the kidney damage remains unproven and warrants further study. Moreover, we cannot exclude the contribution of the MAT—despite its lower inflammation and lack of collagen production—to a systemic inflammatory and profibrotic environment. Second, the pathophysiology of DKD is complex and multifactorial; in clinical practice, kidney dysfunction may be also promoted by preexisting kidney damage [8] and other comorbidities, e.g., arterial hypertension [57]. We thus addressed this in our study by using a model with preexisting kidney and CV damage before diabetes induction; however, further experiments would be needed to define the role of the PRAT in models in which DKD is a consequence of diabetes alone.

In conclusion, the induction of short-term diabetes in MWF rats with established CKD was observed to lead to increased glucose intolerance, insulin resistance, further kidney damage, and cardiac hypertrophy with a preserved ejection fraction. These are the early changes that occur before further increases in albuminuria, hypertension, or arterial stiffness. Additionally, they are associated with proinflammatory and profibrotic alterations in the PRAT; this might contribute to the progression of kidney damage in a paracrine way. Further studies will be necessary to provide in-depth characterizations of the contribution of the alterations that were related to DKD—i.e., the amount of modification that occurred in the advanced glycation end products—and its relationship to alteration in the PRAT.

## 4. Material and Methods

### 4.1. Animals and Diabetes Induction

Sixteen-week-old male MWF rats (Charité, University Medicine Berlin, Germany; n = 20) and aged-matched Wistar rats (Janvier, Nantes, France; n = 5) were housed in individual cages under controlled dark–light cycles (12 h/12 h) and temperature conditions, with free access to food and water for the duration of the study. Only male rats were used, as female rats do not develop the albuminuria phenotype [28].

One group was fed standard chow (MWF-C; n = 10; kcal/g = 2.9), whereas another group received a high-fat, high-sucrose diet (HF/HS; TD08811 ENVIGO, Barcelona, Spain; kcal/g = 4.7; Table 3) for 6 weeks (MWF-D; n = 10). Wistar rats fed normal chow were used as the control for MWF-C rats.

At the start of the HF/HS diet, a low dose of STZ (Sigma Aldrich, Madrid, Spain; 15 mg/kg) was administered i.p. in 6-h-fasted animals to reach blood glucose values above 200 mg/dL. The administration of STZ (15 mg/kg i.p.) was repeated a maximum of 3 times if glycemia was below 200 mg/dL. MWF-C rats received as many vehicle (citrate buffer 0.1 M, pH = 4.5) injections as their littermates.

The optimal dose of 15 mg/kg STZ to achieve blood glucose values above 200 mg/dL in the MWF strain was calculated in a previous pilot study. A dose of 50 mg/kg is commonly used in both Sprague Dawley (SD) rats [29] and C57BL6J mice [58]. However, this dose proved to be highly aggressive and damaging in the MWF strain, in which blood glucose levels above 500 mg/dL were detected, accompanied by a 20% body weight loss in 6 weeks and a high incidence of diarrhea. Since the MWF strain seems to be very sensitive to STZ, we decided to administer increasing STZ doses starting at 5 mg/kg to determine the optimal dose at which the desired hyperglycemia (>200 mg/dL) would be achieved, which turned out to be 15 mg/kg.

As STZ induces renal tubular damage at high doses [55], we included a group treated with STZ plus insulin (MWF-D-ins, n = 3) and fed with standard chow to discriminate between a possible tubular damage induced by STZ itself or by hyperglycemia. This group received the same number of 15 mg/kg STZ injections as their littermates plus a daily insulin (insulin glargin, Lantus Solostar, Frankfurt am Main, Germany) administration for 6 weeks; this treatment comprised as many units (1–3 IU) as necessary to maintain normoglycemia (<120 mg/mL). Glucose levels in this group were monitored daily. At week 6, after urine collection and hemodynamic measurements, the animals were euthanized, blood was collected in heparinized tubes, and tissues (heart, kidney, and perirenal adipose tissue—PRAT, adjacent to kidneys, and MAT) were removed and frozen for study.

All experimental procedures were approved by the Animal Facility of the Instituto Pluridisciplinar (ES-2809-0000197) and the School of Medicine (ES-280790000086; PROEX 205/18), as well as by Institutional Animal Care and Use Committee in accordance with the guidelines for the ethical care of experimental animals of the European Community. Experiments were performed at Instituto Pluridisciplinar. Every effort was made to avoid animal suffering in accordance with the ARRIVE guidelines (Animal Research: Reporting of In Vivo Experiments [59]).

### 4.2. Glucose Determination and Intraperitoneal Glucose Tolerance Test (GTT)

Postprandial blood glucose was determined at 8:30 a.m. just before euthanizing the animals, which had access to food and water during the night before. Fasting blood glucose was monitored once a week in tail blood with a Contour XT glucometer (Barcelona, Spain) after 6 h of fasting. The GTT was performed the last week of dietary treatment. Rats were fasted for 6 h before glucose load (i.p. bolus of 2 g/kg at time 0). Blood glucose level was measured immediately at 0, 15, 30, 45, 60, 90, and 120 min after injection. At the indicated times, blood samples were drawn from tail vein of conscious rats for glucose determination with a Contour XT glucometer (Barcelona, Spain). Insulin levels were determined in plasma samples obtained 15 min after glucose injection by means of a specific enzyme immunoassay kit (Mercodia, Uppsala, Denmark) (2.2% intra-assay variation, 4.9% inter-assay variation). The HOMA-IR (Homeostatic Model Assessment for Insulin Resistance) index was calculated with the following formula: [fasting blood glucose (mmol/L) × fasting plasma insulin (µU/mL)]/22.5. Urine glucose was determined using the glucose Trinder method (Roche Applied Science, Barcelona, Spain).

### 4.3. Determination of Urinary Albumin Excretion and Hemodynamic Parameters

Urine was collected placing the animals in metabolic cages for 24 h after a 1-day adaptation period and urinary albumin excretion (UAE) and creatinine clearance were determined as previously described [26,57].

On the last day, both carotid and femoral arteries were catheterized under anesthesia (80 mg·kg^−1^ ketamine hydrochloride and 12 mg·kg^−1^ xylazine hydrochloride, i.p.) and blood pressure waves were recorded in a PowerLab system (ADInstruments, Oxford, UK) to calculate both systolic (SBP) and diastolic blood pressure (DBP) [23,24]. Pulse wave velocity (PWV) was calculated using the following formula [23,24]: D (meters)/Δt (seconds), where D is the distance between carotid and femoral arteries and where Δt is the time delay, determined using the displacement of the two pressure waves (carotid and femoral).

### 4.4. Acquisition and Analysis of Cardiac Structure and Function by Magnetic Resonance Imaging

Magnetic resonance imaging (MRI) was performed with a Biospec BMT 47/40 spectrometer (Bruker, Ettlingen, Germany) operating at 4.7-Teslas and equipped with a 12 cm gradient system. Animals were anesthetized with a mixture of 3% isoflurane in oxygen (1.5 L/min for induction and 1.0–1.5% at 1.0 L/min for maintenance). Body temperature was maintained at 36 °C. Heart rate and respiration were monitored and used to trigger image acquisition with 1025 SAM monitoring and gating system (SA Instruments, Inc., New York, NY, USA). Several gradient echo images with different orientations were acquired to localize the short axis planes. Images were cardiac and respiratory triggered and up to seven slices were acquired in a cardiac cycle. Repetition time (TR) was variable depending on the animal’s heart and respiration rate. Other imaging parameters were as follows: echo time (TE) = 2.7 ms; flip angle (θ) = 80°; field of view (FOV) = 6 × 6 cm^2^; slice thickness = 2.0 mm; matrix size = 128 × 128; number of averaged images = 2. Once the short axis was set, a multi-slice white blood CINE sequence was used to image the rat’s entire heart. For these experiments, a cardiac- and respiratory-triggered FLASH sequence was used. Ten images per cardiac cycle were acquired to completely cover the cardiac cycle. Other imaging parameters were as follows: TE = 2 ms; θ = 80°; FOV = 5.12 × 5.12 cm^2^; slice thickness = 1.5 mm; matrix size = 128 × 128; number of averaged images = 8. A total of 8–10 slices were acquired to cover the whole heart. The data were transferred to a PC for analysis, which was carried out with ImageJ 1.49v (NIH, Bethesda, MD, USA). For each slice of the CINE sequence, the left ventricle (LV) was segmented in the images corresponding to diastole and systole. The LV volume for each slice was summed to obtain the total left ventricular volume at the end of diastole and systole (LVEDV and LVESV, respectively). The heart rate for each rat was calculated as the mean value of the heart rate during the entire CINE experiment. The ejection fraction (EF), cardiac output (SV), and cardiac output (CO) were obtained from these data.

### 4.5. RNA Extraction and Real-Time PCR (RT-qPCR)

Total RNA was isolated from the kidney, heart, PRAT, and MAT using Qiazol Reagent (Qiagen, Düsseldorf, Germany). The samples were processed using RNA Spin illustraTM (GE Healthcare, Chicago, IL, USA). The concentration and purity of RNA were assessed with NanoDropTM 2000/c (Fisher Scientific, Pittsburgh, PA, USA). Reverse transcription was performed on 500 ng of RNA with iScript cDNA synthesis kit (BioRad, Hercules, CA, USA) using random hexamer primers. Optimal annealing temperature and amplicon sizes were checked for each pair of primers. RT-qPCR analyzes were performed in a CFX96 Instrument (BioRad, Hercules, CA, USA). A measure of 10.00 ng of cDNA from ten samples of each group were run in duplicate and the mRNA levels were determined using intron-skipping primers with *Gapdh*, *Tbp*, and *Atpaf-1* as housekeeping genes and SYBR Green Master Mix (Applied Biosystems, Foster City, CA, USA). All primer sequences are detailed in Table 4.

### 4.6. Statistical Analysis

The number of animals per group was calculated to reach a significance level of 5% (*p* < 0.05), with a required power of 80%, a difference of 1.6 typical deviation to be detected, and 10% sample loss. Continuous variables were compared by Student’s *t* test or one-way ANOVA followed by a Dunnett test comparing MWF-D with the (non-diabetic) MWF group as control. Categorical variables were compared using Fisher’s exact test. Data analysis was performed with GraphPad Prism 8 for animal data. Data are presented as mean ± standard error of the mean and statistical significance was considered for *p* < 0.05.

## Figures and Tables

**Table 1:** Food intake, body, and tissue weights.

	Wistar	MWF-C	MWF-D
Final weight (g)	557.4 ± 20.5	400.3 ± 9.6 *	433.0 ± 4.4 #
Food intake (kcal/day)	44.15 ± 2.4	47.95 ± 2.0	68.7 ± 6.8 #
PRAT weight/tibia length (g/mm)	0.18 ± 0.02	0.09 ± 0.01 *	0.15 ± 0.01 #
MAT weight/tibia length (g/mm)	0.13 ± 0.02	0.07 ± 0.01	0.12 ± 0.01 #
Fasting glucose (mg/dL)	130.5 ± 11.1	124.6 ± 3.9	243.0 ± 28.4 #
Post-prandrial glucose (mg/dL)	291.8 ± 49.6	302.3 ± 24.7	481.2 ± 33.1 #
Fasting insulin (ng/mL)	3.3 ± 0.3	2.6 ± 0.4	3.4 ± 0.4
Post-prandrial insulin (ng/mL)	8.3 ± 0.9	5.8 ± 0.3 *	3.4 ± 0.4 #

PRAT, perirenal adipose tissue; MAT, mesenteric adipose tissue. Data are presented as mean ± SEM. * *p* < 0.05 compared to Wistar; # *p* < 0.05 compared to MWF-C.

**Table 2:** Hemodynamic parameters.

	Wistar	MWF-C	MWF-D
**SBP (mmHg)**	97.3 ± 5.6	146.2 ± 6.3 *	147.6 ± 11.3 *
**DBP (mmHg)**	71.16 ± 4.9	106.1 ± 4.8 *	114.7 ± 10.1 *
**PWV (m/s)**	5.9 ± 0.2	8.1 ± 0.5 *	8.2 ± 0.8 *

Data are presented as mean ± SEM. SBP, systolic blood pressure; DBP, diastolic blood pressure; PWV, pulse wave velocity; * *p* < 0.05 compared to W.

**Table 3:** Composition of diets.

	Standard Chow (C)		High-Fat, High-Sucrose (HF/HS) Diet	
	Composition (%)	% kcal	Composition (%)	% kcal
Fat of animal origin	10	12	23	45
Carbohydrate	70	68	48	40
Protein	20	20	17	15
Other	-	-	20	3

**Table 4:** Primer sequences.

Gene	Accession Number	Forward (5′-3′)	Reverse (5′-3′)
Rn *Kim-1*	NM_173149.2	ATTGTTGCCGAGTGGAGAT	TGTGGTTGTGGGTCTTGTAGT
Rn *Ngal*	NM_130741.1	GGCCGACACTGACTACGACC	GCCCCTTGGTTCTTCCGTAC
Rn *At1r*	NM_030985.4	GTTGGGAGGGACTGGATGATGC	TGATCGGGTGGAACAGGACTCA
Rn *At2r*	NM_012494.4	CCTCTGGAAAGCTGGCAAGTGT	TATTCGCTCTGTCCACTGGGGA
Rn *Col1a1*	NM_053304.1	GGATGCCATCAAGGTCTACTGC	TGAGTGGGGAACACACAGGTCT
Rn *IL-1β*	NM_031512.2	TGACAGACCCCAAAAGATTAAGGA	CGAGATGCTGCTGTGAGATTTG
Rn *IL-6*	NM_012589.2	CCTGGAGTTTGTGAAGAACAACTT	TGGAAGGTGGGGTAGGAAGGAC
Rn *IL-10*	NM_012854.2	CAGTGGAGCAGGTGAAGAATGA	CATTCATGGCCTTGTAGACACC
Rn *Tnf-α*	NM_012675.3	CTACTGAACTTCGGGGTGATCG	GGCTTGTCACTCGAGTTTTGAGA
Rn *Tgf-β*	NM_021578.2	ATGGTGGACCGCAACAAC	CAGCAATGGGGGTTCTGG
Rn *Anp*	NM_012612.2	ATACAGTGCGGTGTCCAACA	CGAGAGCACCTCCATCTCTC
Rn *Bnp*	NM_031545.1	TCCTTAATCTGTCGCCGCTG	TTTTCTCTTATCAGCTCCAGCA
Rn *Gapdh*	NM_017008.4	AAGGCTGAGAAATGGGAAGCTC	CCATTTGATGTTAGCGGGATCT
Rn *Atpaf-1*	NM_001107959.1	GATCTCTCCAAGAAGCTGCAAG	AAGATGACCCCAAGGCATTTTT
Rn *Tbp*	NM_001004198.1	GACCCACCAGCAGTTCAGTAGC	CAATTCTGGGTTTGATCATTCTG

## Data Availability

The data supporting the findings of this study are available from the corresponding author upon reasonable request.
